# Use of botulinum toxin in the management of dystonia in Parkinson’s disease

**DOI:** 10.3389/fnins.2024.1371601

**Published:** 2024-04-08

**Authors:** Charenya Anandan, Joseph Jankovic

**Affiliations:** ^1^Department of Neurology, Parkinson's Disease Center and Movement Disorders Clinic, Baylor College of Medicine, Houston, TX, United States; ^2^Neurology Care Line, Michael E. DeBakey VA Medical Center, Houston, TX, United States

**Keywords:** dystonia, Parkinson’s disease, botulinum toxin, blepharospasm, cervical dystonia

## Abstract

Botulinum toxin is one of the most potent neurotoxins, but when injected into an overactive muscle, it can transiently alleviate an involuntary movement, such as dystonia. The primary aim of this article is to provide a comprehensive review of the various forms of dystonia observed in patients with Parkinson’s disease who can benefit from a therapeutic trial of botulinum toxin. Although most of these indications are not supported by randomized controlled clinical trials and, therefore, not approved by the Food and Drug Administration, there are many open-label trials supporting a large body of empirical experience testifying to the benefits of botulinum toxin treatment in these conditions.

## 1 Introduction

Dystonia is present in up to 30% of patients with Parkinson’s disease (PD) (Anandan and Jankovic, 2023; Matar and Bhatia, 2023). In some cases of PD or other parkinsonian disorders, dystonia may be one of the motor features, and in other cases, dystonia may be the dominant movement disorder with parkinsonism as the secondary phenomenon, classified as “‘combined’ dystonias” (Albanese et al., 2013). The form of dystonia can be a clue into the type of parkinsonism the patient has. For example, retrocollis/truncal extension is commonly seen in progressive supranuclear palsy (PSP), whereas anterocollis, Pisa sign, and levodopa-induced dyskinesia in the form of oromandibular dystonia are typically seen in multiple system atrophy (MSA), and asymmetric limb dystonia is often present in patients with corticobasal syndrome (CBS) (Vanek and Jankovic, 2001).

The non-aerobic bacteria *Clostridium botulinum* produces botulinum toxin (BoNT), the most potent biologic toxin (Anandan and Jankovic, 2023). BoNT acts by blocking the release of acetylcholine at the neuromuscular junction, thus causing muscle weakness. This property has been used for therapeutic purposes, including in the treatment of dystonia. There are many indications for BoNT in patients with PD and other parkinsonian disorders (Jankovic, 2009; Cardoso, 2018; Jost, 2021; Lapostolle et al., 2022; Anandan and Jankovic, 2023; Jabbari and Comtesse, 2023). This article will focus on the use of BoNT in PD-associated dystonia.

## 2 Method

On 11 December 2023, we conducted a PubMed advanced search with the title words “botulinum,” and “Parkinson” and the text word “dystonia.” This yielded 14 articles. We also conducted a PubMed advanced search with the title words “botulinum” and “parkinsonism” and the text word “dystonia.” This yielded eight articles. This article was formulated using these articles and other relevant articles.

## 3 Discussion

There are numerous forms of dystonia in patients with PD and other parkinsonian disorders that can benefit from BoNT (Table 1).

**Table 1:** Treatment of PD-related dystonia with BoNT: review of the literature.

	Location of dystonia	Formulation	Number of patients	Dose and muscles	Results	Adverse event (AE)
1.1	Limb dystonia					
	Gupta and Visvanathan (2016)	OnaBoNTA	6 PD pts. with DBS and off foot dystonia	250–400 U total- selected based on posture (with 50–150 per TP, FDB, FHL, FDL, GS, EHL)	3 weeks after injection significant improvement noted in pain and dystonia	None
	Gupta et al. (2018)	OnaBoNTA	14 pts. with foot dystonia (11 with PD)	Affected muscles injected (FHL, FDL, FDB, GS, TP) were injected with 100–300 U	Stride and step length increased *p* = 0.02 in the affected limb	None
	Pacchetti et al. (1995)	OnaBoNTA	30 PD pts. with off foot dystonia were injected	TA, GS, TP, FDL, EHL (selected based on posture) with a median dose of 40 U per muscle in 2 sites (median dose of 70 U per pt.)	In 21 pts., pain resolved at 4 months	None
	Rieu et al. (2018)	IncoBoNTA	IncoBoNTA or placebo was injected in 45 parkinsonian pts	Group 1: 100 U in FDB, placebo in FDL Group 2: 100 U in FDL, placebo in FDB Group 3: placebo in FDL and FDB	Mean clinical global impression change (CGI) in the BoNT group compared to the placebo was *p* = 0.039	Localized foot pain (1) Transient loss of sensation in the leg (1) Falls (9)
	Kurtis et al. (2008)	OnaBoNTA and RimaBoNTB	8 pts. had EHL dystonia due to varied pathologies of which 1 had wearing-off dystonia	Final stable dose was 50 U in EHL in the PD pt	EHL injection doses were found to be safe, even up to 160 u OnaBoNTA	None
	Lindholm et al. (2017)	IncoBoNTA	10 PD pts. with striatal foot deformity were injected	10-25 U in EHL, 20-50 U in FDL, 40–70 TP, 15 U in GM	Standing balance improved at 4 weeks but not at 16 weeks	None
	Giladi et al. (1994)	OnaBoNTA	2 pts. with striatal toe	50–70 U in EHL	80–90% subjective improvement	Some EHL weakness
	Cordivari et al. (2001)	AboBoNTA	14 pts. with a clenched fist (of which 7PD, 3 CBD)	220–1,200 units in different affected muscles	All 14 had improvement in posture; 4 of 7 PD pts. had meaningful improvement in function	None
	Ni et al. (2023)	BoNT-A (HengLi, Lanzhou institute of biological products Co. LTD, China)	25 PD pts. with foot dystonia	Various doses in TP, TA, FHL, FDL, FDB, EHL, GM, FHB approximately 85–250 U per limb	BoNT reduced pain and helped posture	Transient weakness (3) Fullness sensation (1)
1.2	Blepharospasm					
	Martinez-Ramirez et al. (2014)	OnaBoNTA, RimaBoNTB	64 (41 had primary) and 23 had secondary BSP (of which 12 pts. had PD and 3 had PSP)	39.2–57.2 units in lateral oculi, pretarsal OO, corrugator, procerus, and frontalis	Both primary and secondary BSP responded well	Diplopia (3) Ptosis (1) Dry eyes (1) Bruising (1)
	Lepore et al. (1995)	OnaBoNTA	1 pt. with BSP	20 U in orbicularis oculi	Improvement for 2–3 months	None
1.3	Oromandibular dystonia					
	Agarwal (2019)	OnaBoNTA	2 PD pts. with wearing off jaw-opening dystonia	10 U each into lateral pterygoid and digastric muscles	Improvement noted in OMD	None
	Rosales et al. (2011)	AboBoNTA	109 XDP pts. injected (50 OMD, 35 lingual, 24 truncal axial)	Varied based on pathology	Substantial improvement was noted on the dystonia rating scale (DRS) with OMD, and moderate improvement noted with truncal dystonia	Dry mouth and dysphagia were the most common complications
1.4	Trunk abnormalities					
	Bonanni et al. (2007)	AboBoNTA	9 pts. with lateral axial dystonia in dopa-responsive parkinsonism- double-blind cross-over trial of BoNT vs. placebo	Total of 500 U injected into the paraspinal muscle	BoNT effective in 6 pts	None
	Artusi et al. (2019)	OnaBoNTA	15 PD pts. with Pisa syndrome	Mean of 50–75 units per paraspinal muscle and 25–50 U per abdominal muscle	11/13 pts. improved in lateral flexion	None
	Tassorelli et al. (2014)	IncoBoNTA	26 PD pts. were enrolled in the randomized placebo-controlled trial for Pisa Syndrome	50-200 U injected in some of the following muscles: rectus abdominus, inferior thoracic paraspinal, iliopsoas, multifidus	Lateral flexion benefited the treatment group (*p* = 0.044)	None
	Azher and Jankovic (2005)	OnaBoNTA	Of 16 camptocormia pts. injected (11 with PD and 4 with axial dystonia)	300–600 U injected in rectus abdominis of 9 pts. (contraction of rectus abdominis felt)	4 of 9 had improvement in symptoms	None
	Fietzek et al. (2009)	IncoBoNTA	10 pts. of camptocormia (does not specify if in PD)	100–300 units injected in iliopsoas or rectus abdominis (active abdominal contractions not reported)	No improvement was reported	Soreness in muscle (2)
	von Coelln et al. (2008)	AboBoNTA	4 pts. with camptocormia (3 PD and 1 MSA)	500-1500 U per iliopsoas injected	2 experienced modest benefits, 2 experienced worsening symptoms	Pruritis at injection site (1), hip flexion weakness (4)
	Todo et al. (2018)	OnaBoNTA	6 PD pts. with camptocormia	75 to 90 U injected into the external oblique	Subjective relief noted in 4 of 6 pts	None

### 3.1 Limb dystonia

Foot dystonia, produced by involuntary, repetitive, twisting contractions of muscles in the foot, occurs most often when carbidopa-levodopa wears off (Jost, 2021; Anandan and Jankovic, 2023). This “wearing off” foot dystonia is usually characterized by ankle plantarflexion/inversion, with toes curling usually downward, but sometimes the toes (particularly the big toe) extend. There are several reports that have discussed the benefits of BoNT in “wearing off” foot dystonia (Gupta and Visvanathan, 2016; Rieu et al., 2018). In our clinic, we treat this form of dystonia with BoNT injections in the tibialis posterior, flexor digitorum brevis, flexor digitorum longus, and extensor hallucis longus. We occasionally also target the gastrocnemius if there is troublesome plantar flexion or associated calf cramps. Among 13 patients with upper limb dystonia due to X-linked dystonia parkinsonism (XDP), a median 12-week benefit after BoNT injection was noted, with 15% of patients experiencing weakness post-injection (Rosales et al., 2011).

“Striatal toe” is a form of fixed deformity, which is different from “wearing off” foot dystonia. Approximately 13% of patients with PD can have striatal limb deformities and the cause remains unknown (Wijemanne and Jankovic, 2019). This can affect gait and reduce the ability to stand and ambulate (Lindholm et al., 2017). Targeting the extensor hallucis longus with BoNT can be quite effective (Kurtis et al., 2008).

Clenched fists due to dystonia can be a late complication in PD and other parkinsonism disorders (Jocson and Lew, 2019). There are reports of this focal dystonia improving with BoNT, thus improving function and facilitating good hygiene (Cordivari et al., 2001). Striatal hand and foot deformities are not thought to derive from dystonia; hence, BoNT has not been traditionally used to treat these conditions (Wijemanne and Jankovic, 2019).

### 3.2 Cervical dystonia

Cervical dystonia (CD) is the most common form of focal dystonia encountered in movement disorders clinics. It can lead to abnormal head/neck posture and movement, tremors, and pain (Albanese et al., 2023). CD was found to be present in 33.9% of patients with PD (Jost, 2021). Oromandibular dystonia, anterocollis, and scoliosis (Pisa sign) are commonly seen in patients with parkinsonism, especially in those with MSA (Marsili and Truong, 2020). Dysphagia is a common complication of anterocollis, and this can be worsened with anterior neck muscle BoNT injections, particularly with bilateral injections; hence, it is crucial to start with low doses of BoNT in these instances. The usefulness of BoNT in the treatment of CD has been demonstrated in numerous trials (Hammoud and Jankovic, 2022). In one study of 144 patients with CD and 24 due to parkinsonism, the duration of benefit, dosage of BoNT, and occurrence of dysphagia after BoNT were similar in patients with CD and associated parkinsonism vs. CD without parkinsonism (Patterson et al., 2016). Of the 109 patients with XDP, 21 had torticollis, 11 had retrocollis, 14 had laterocollis, and 10 had anterocollis (Rosales et al., 2011). The median dose of abobotulinum toxin A injected per visit varied from 250 to 500 units. The authors reported improvement lasting approximately 12 weeks; the frequency of dysphagia ranged from 14% for torticollis to 20% for anterocollis (Rosales et al., 2011).

### 3.3 Blepharospasm

Blepharospasm is a form of focal dystonia that is characterized by involuntary eye closure due to contractions of the orbicularis oculi and other periorbital muscles (Romano et al., 2022). Blepharoclonus, manifested by repetitive spasms of the eyelids without actual eye closure, was noted in 84% of patients with PD in one recent study (Margolesky et al., 2024). Apraxia of eyelid opening, which is manifested by the inability to open eyes (presumably by inhibition of the levator oculi), typically associated with compensatory frontalis contraction, can also be seen in PD but is even more frequent in patients with atypical parkinsonism (Piccione et al., 1997; Yoon et al., 2005). A retrospective review of 64 patients with blepharospasm, 41 with primary blepharospasm, and 23 with secondary blepharospasm, of which 12 had PD and 3 had PSP, examined the effects of BoNT treatment. BoNT injections (either onabotulinumtoxin A or rimabotulinumtoxin B was used) into the periorbital muscles (including the lateral oculi, pretarsal, procerus, corrugator, and frontalis) resulted in benefits lasting 9.43 and 9.67 weeks in primary and secondary blepharospasm, respectively (Martinez-Ramirez et al., 2014). Onabotulinumtoxin A (12.5 units per eye injection into the junction of the preseptal and pretarsal portions) can produce relief in patients with apraxia of the eyelid opening associated with parkinsonism (Piccione et al., 1997). In another study, onabotulinumtoxin A 20 U into the pretarsal portion of orbicularis oculi produced benefits for 2–3 months (Lepore et al., 1995). There are additional reports of improved apraxia of eyelid opening after BoNT (Defazio et al., 1990; Jankovic, 1996).

### 3.4 Oromandibular dystonia

Oromandibular dystonia is relatively uncommon in PD, but it is a relatively common complication of levodopa therapy in MSA (Anandan and Jankovic, 2023). Wearing off jaw dystonia has been reported to improve after BoNT injections (Agarwal, 2019). In X-linked dystonia parkinsonism, 32 patients had jaw-opening dystonia, treated with BoNT injections into the lateral pterygoid and anterior digastric muscles with a median dose of 100 U of abobotulinumtoxin A. A total of 12 patients with jaw closing dystonia (injected into masseter, temporalis, and medial pterygoid with a median dose of 150 U of abobotulinumtoxin A) and 6 patients with jaw deviation (injected into lateral pterygoid and temporalis with a median dose of 100 U abobotulinumtoxin A) noted median improvement lasting 8–16 and 12–24 weeks, respectively; 17–19% experienced adverse effects such as dry mouth and dysphagia (Rosales et al., 2011). Many patients with PD and other parkinsonian disorders experience temporomandibular joint pain, most likely related to daytime or nocturnal bruxism (Ondo et al., 2018; Verhoeff et al., 2022; Minervini et al., 2023). Based on a double-blind, placebo-controlled trial, BoNT injections targeting the masseter and temporalis muscles have been found to be safe and effective in the treatment of bruxism (Ondo et al., 2018).

### 3.5 Laryngeal dystonia

Spasmodic dysphonia is a focal dystonia involving the laryngeal muscles associated with either adductor spasm (resulting in a strained voice and breaks in phonation) or, rarely, abductor spasm (associated with breathy voice and voiceless pauses) or both (Truong and Bhidayasiri, 2006; Simonyan et al., 2021). These can be successfully treated with BoNT injections into the thyroarytenoid muscle (for adductor spasmodic dysphonia) and the posterior cricoarytenoid muscle (for abductor spasmodic dysphonia). In one patient with PD who did not respond to BoNT-A (abobotulinumtoxin A followed by onabotulinumtoxin A was tried) for spasmodic dysphonia, BoNT-B (Neurobloc) was effective (Sachdev et al., 2019). It is unclear why there was no response to type A BoNT in this spurious single-case report.

### 3.6 Trunk abnormalities

One-third of PD patients have abnormal truncal postures, which may also include Pisa syndrome (leaning to one side due to scoliosis), lateral axial dystonia, and camptocormia (Wijemanne and Jankovic, 2019). Pisa syndrome occurs in 7–10% of PD patients (Artusi et al., 2019). An improvement in the axial posture was found in a double-blind placebo-controlled trial of incobotulinum toxin A injected into muscles individually identified for patients with Pisa syndrome (Tassorelli et al., 2014). A PD patient with Pisa syndrome improved after undergoing paraspinal muscle abobotulinumtoxin A injections and a rehabilitation program (Santamato et al., 2010). Pisa syndrome due to lateral axial dystonia (dystonic scoliosis) can be painful and affect mobility (Bonanni et al., 2007). Camptocormia is characterized by moderate to marked flexion of the thoracolumbar spine caused by isolated axial dystonia or complex dystonia related to underlying PD or other parkinsonian disorders (Azher and Jankovic, 2005; Wijemanne and Jankovic, 2019). Several case series have reported inconsistent improvement of camptocormia with BoNT injections, but BoNT targeting the rectus abdominis or external oblique muscle may be very effective in improving the axial posture (Azher and Jankovic, 2005; Colosimo and Salvatori, 2009; Fietzek et al., 2009). Among 109 patients with X-linked dystonia parkinsonism, 12 had trunk flexor dystonia, 7 had trunk extensor dystonia, and 5 had lateral trunk dystonia, and they were injected with abobotulinumtoxin A with a median dose of 400 U in rectus abdominis, 750 U in bilateral erector spinae, and 1,000 U in ipsilateral erector spinae, respectively (Rosales et al., 2011).

### 3.7 Levodopa-related dystonia

Levodopa-related dystonia is a well-recognized complication of levodopa therapy in patients with PD (Jameson, 1970; Cooper, 1972; Pfeiffer and LeDoux, 2015). One single case report described a patient with PD who developed jaw deviation dystonia during a peak dose of levodopa, which improved after medication reduction and onabotulinum toxin A injection of 40 U into each lateral pterygoid and 7.5 U into each submental muscle (Pfeiffer and LeDoux, 2015). In another PD patient with jaw-opening dystonia, a 40% improvement was noted after BoNT was injected into lateral pterygoids (Tee et al., 2019). The use of BoNT for levodopa-related dystonia is based on anecdotal case reports and our own experience. We have found that BoNT injections into the affected muscles, particularly those involved in wearing off-foot dystonia, are very effective in most patients. It is best for these conditions to be treated by movement disorder-trained neurologists who have excellent understanding and experience in BoNT use.

Orofacial dyskinesia, often in the form of oromandibular dystonia (discussed above), raises concern for MSA (Onder and Comoglu, 2023). In a retrospective chart review of 83 patients with PSP, 3 patients with levodopa-induced dyskinesias were identified (1 blepharospasm, 1 jaw closure dystonia, and 1 limb dystonia), testifying to the rare occurrence of this complication in patients with PSP (Barclay and Lang, 1997). This is also suggested by rare case reports of oromandibular dystonia (Tan et al., 2003; Modreanu et al., 2018), facial dystonia (Chung and Kim, 2012), and cranial dystonia (Onder and Comoglu, 2023) in PSP that resolved after discontinuation of levodopa.

## 4 Conclusion

BoNT usage is expanding across many fields of medicine and beyond. Isolated dystonia continues to be one of the most common indications for BoNT treatment, but BoNT is increasingly used in patients with combined and complex dystonia in the setting of PD and other parkinsonian disorders. Although most of these forms of dystonia are not yet supported by randomized controlled clinical trials, their improvement with BoNT is becoming well established. BoNT is also used for many other PD-related symptoms, such as tremors, sialorrhea, dyskinesia, urinary symptoms, and others. Patients receiving BoNT in multiple locations should have the injections administered on the same day, and the total dose of BoNT should be kept below 600 units to reduce the risk of generalized weakness or immunogenicity.

## Author contributions

CA: Conceptualization, Investigation, Methodology, Writing – original draft, Writing – review & editing. JJ: Methodology, Supervision, Writing – original draft, Writing – review & editing.

## Figures and Tables

**Table:** 

aboBoNTA	Abobotulinumtoxin A
ALO	Apraxia of eyelid opening
BSP	Blepharospasm
BoNT	Botulinum toxin
EMG	Electromyography
EHL	Extensor hallucis longus
FDB	Flexor digitorum brevis
FDL	Flexor digitorum longus
FHB	Flexor hallucis brevis
FHL	Flexor hallucis longus
GM	Gastrocnemius
GS	Gastrocnemius-soleus
incoBoNTA	Incobotulinumtoxin A
MSA	Multiple system atrophy
OO	Orbicularis oculi
OMD	Oromandibular dystonia
OnaBoNTA	Onabotulinumtoxin A
PSP	Progressive supranuclear palsy
PD	Parkinson’s disease
Pt, Pts	Patient, patients
RimaBoNTB	Rimabotulinumtoxin B
TA	Tibialis anterior
TP	Tibialis posterior
U	Units
XDP	X-linked dystonia parkinsonism
